# Supplementary material for: Ancient DNA Methods Improve Forensic DNA Profiling of Korean War and World War II Unknowns
Source: Genes (Basel). 2022 Jan 11;13(1):129. doi: 10.3390/genes13010129 (PMC8774965; doi:10.3390/genes13010129)
Supplement: Supplementary file 1 [file genes-13-00129-s001.zip › Supplementary Figures S1-S4.pdf]

Supplementary Information

SI Section 1: Identification and removal of pig sequences

The distribution of coverage across the mtDNA genome was visually examined for each library that was enriched for human mtDNA during the CLC workflow. During this examination it was observed that the samples and controls that had undergone SRSLY library preparation had unexpected spikes in coverage that also introduced unexpected variants (Figsures S1A and S2A). To determine if this was caused by the mismapping of non-human mtDNA, we ran the data through a pipeline typically used for sediment samples [1] uses BLAST [2] and MEGAN [3] to assign unique sequences to different mammalian families. With this pipeline we found that 11 of the 15 samples libraries contained pig (suidae) mtDNA including the two negative controls (Figure S3). As the pig DNA was present in both the samples and negative controls, this indicates that this contaminant was from the SRSLY library prep kit reagents. To ensure that the presence of contaminant pig mtDNA did not impact downstream processing a “pig out” pipeline was used for processing all SRSLY libraries. For each the MPI and CLC workflows this was based on competitive mapping, which has been shown to be an effective technique for removing mismapped sequences [4]. For the MPI workflow, the sequences for each library were mapped to both the human reference mtDNA genome [5] and a pig (Sus scrofa) mtDNA genome (NC_000845.1 [6]) and only sequences that map better to the human mtDNA than the pig mtDNA were retained. For the CLC workflow, sequences were first quality trimmed (quality limit 0.05) and ambiguous bases were trimmed (ambiguous limit 2). Reads below 30 base pairs in length were removed. The remaining reads were mapped to the Sus scrofa genome with stringent mapping parameters (length fraction 0.85 and similarity fraction 0.95). Any unmapped sequences were then mapped to the rCRS with equally stringent mapping parameters. Mapping to the rCRS and all subsequent steps in the PIGout workflow were identical to those in the previously mentioned CLC workflow used to analyze all other libraries. After this processing the coverage spikes and unexpected variants were no longer present (Figures S1B and S2B).

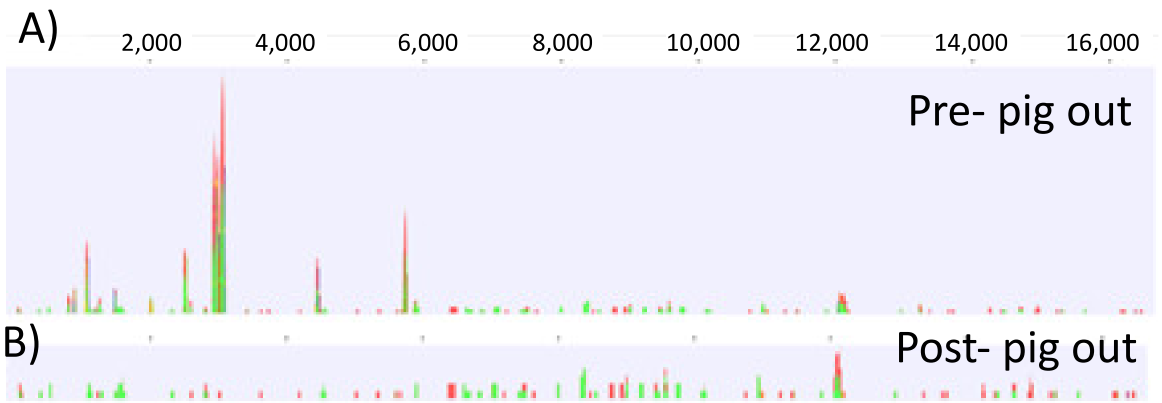

Supplementary Figure S1. The coverage distribution across the mtDNA genome for a SRSLY library negative control before (**A**) and after (**B**) CLC pig out workflow. Green portion represents forward reads and red portion represents reverse reads

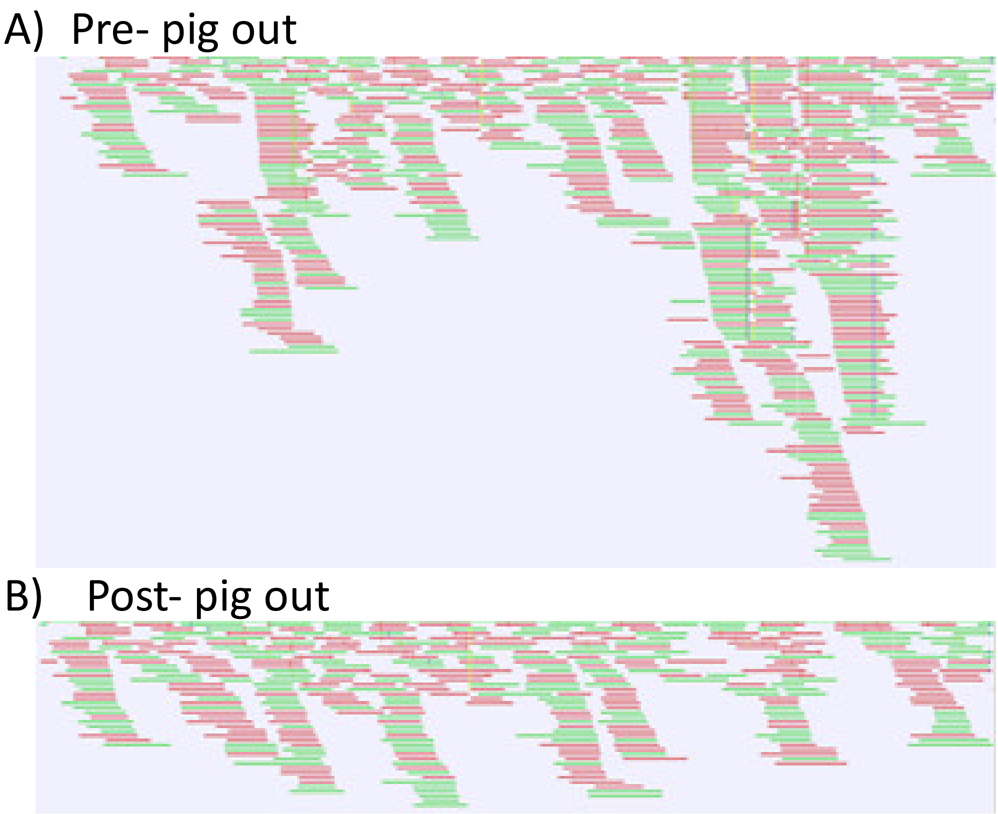

Supplementary Figure S2. Sequences mapped to positions 2300-3200 of the human mtDNA reference genome from a SRSLY library (sample 0378) pre- (**A**) and post- (**B**) CLC pig out workflow. Horizonal green lines represent individual forward reads and red lines reverse reads. Vertical lines represent differences from the reference (blue = C, yellow = G, red = A, green = T). A maximum of 100-fold in displayed range for (**A**) and 48-fold for (**B**).

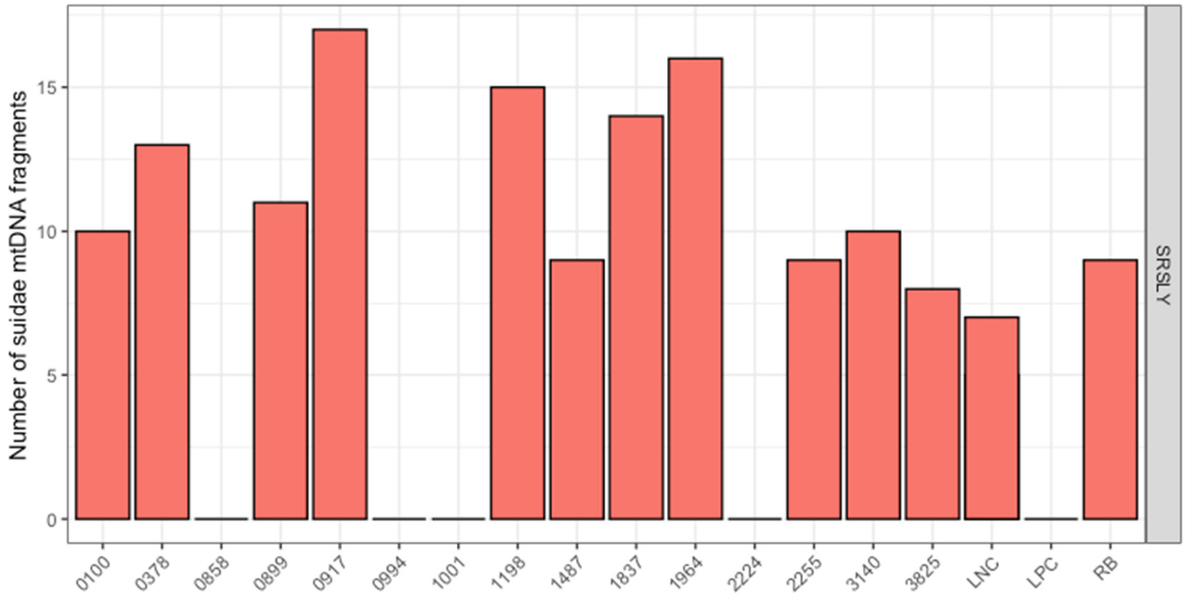

Supplementary Figure S3. The number of mtDNA fragments assigned to the suidae family for samples that underwent SRSLY library preparation. The first fifteen libraries are from samples, followed by positive and negative controls. LNC: library negative control; LPC: library positive control; RB: reagent blank.

SI Section 2: Calculation of relative informative sequences

In order to evaluate the success of the different extraction and library preparation protocols we calculated the informative sequences content after shotgun sequencing as described in Gansauge et al., 2017 [7]. The equation used is:

$$Informative\ Sequences = \frac{\#mapped\ sequences\ \geq 35\ bp}{\#raw\ sequences}\ x\ \#qPCR\ molecules$$

In this equation the number of mapped sequences at least 35 bp is calculated before duplicate removal. This equation calculates the proportion of total sequenced DNA molecules that are informative (i.e., map to the reference genome of interest) and applies it to the number of molecules in the library as determined by pre-amplification qPCR (materials and methods 2.3.4) to determine the total informative sequences in a library.

While this initial calculation is informative when comparing values within a single sample (Figure S4A), it is less useful when comparing across samples. This is because differences between protocols for samples with higher amounts of DNA will be weighted higher than samples with lower amounts of DNA. For this reason, we used relative informative sequences to make comparisons among samples. For this calculation, within each sample, the informative sequences from each protocol is divided by the protocol that resulted in the most informative sequences (Figure S4B). This will place all values between 0 and 1 and allow the comparison of values across samples. We used these relative informative sequences calculation to evaluate the success of different extraction and library preparation protocols.

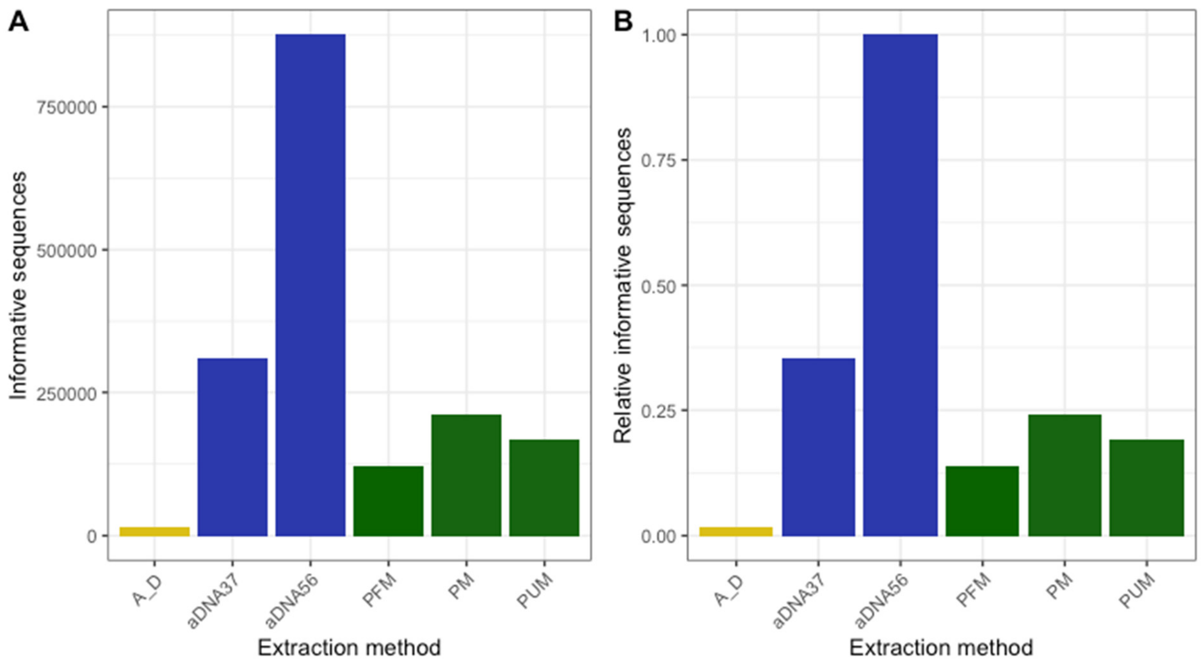

Supplementary Figure S4. The (**A**) informative and (**B**) relative informative sequences recovered from samples 2018H1198-02A2 across six difference extraction protocols. The ancient DNA protocols (aDNA37: 37°C digestion; aDNA56: 56 °C digestion) are colored in blue, forensic protocols (PM: PCIA with Min Elute; PUM: PCIA with Min Elute and USER

treatment; PFM: PCIA with Min Elute and FFPE treatment) in green and the combined forensic ancient DNA protocol in yellow.

References

1. Slon, V., et al., *Neandertal and Denisovan DNA from Pleistocene sediments.* Science, 2017. **356**(6338): p. 605-608.
2. Altschul, S.F., et al., *Basic local alignment search tool.* J Mol Biol, 1990. **215**(3): p. 403-10.
3. Huson, D.H., et al., *MEGAN analysis of metagenomic data.* Genome Res, 2007. **17**(3): p. 377-86.
4. Feuerborn, T.R., et al., *Competitive mapping allows for the identification and exclusion of human DNA contamination in ancient faunal genomic datasets.* BMC Genomics, 2020. **21**(1): p. 844.
5. Andrews, R.M., et al., *Reanalysis and revision of the Cambridge reference sequence for human mitochondrial DNA.* Nat Genet, 1999. **23**(2): p. 147.
6. Lin, C.S., et al., *Complete nucleotide sequence of pig (Sus scrofa) mitochondrial genome and dating evolutionary divergence within Artiodactyla.* Gene, 1999. **236**(1): p. 107-14.
7. Gansauge, M.T., et al., *Single-stranded DNA library preparation from highly degraded DNA using T4 DNA ligase.* Nucleic Acids Res, 2017. **45**(10): p. e79.
